# Modulating the Optical Properties of Initial Caries Lesions Through Low-Viscosity Resin Infiltration: A Spectrophotometric Evaluation

**DOI:** 10.3390/jfb17080423

**Published:** 2026-08-21

**Authors:** Paweł Maksymiuk, Maja Ptasiewicz, Joanna Zubrzycka, Ilona Wójcik-Chęcińska, Katarzyna Sarna-Boś, Piotr Stachurski, Renata Chałas

**Affiliations:** 1 Department of Oral Medicine, Medical University of Lublin, ul. Dra Witolda Chodźki 6, 20-093 Lublin, Poland; 2 Preclinical Dentistry Lab, Medical University of Lublin, ul. Dra Witolda Chodźki 6, 20-093 Lublin, Poland; 3 Department of Dental Prosthetics, Medical University of Lublin, ul. Dra Witolda Chodźki 6, 20-093 Lublin, Poland; 4 Department of Dental Emergency, Medical University of Lublin, ul. Dra Witolda Chodźki 6, 20-093 Lublin, Poland

**Keywords:** nanopores, refractive index, resin infiltration, initial caries lesion, tooth color, spectrophotometry, colorimetry

## Abstract

Objectives: Initial caries lesions (ICLs) are the earliest manifestation of dental caries, characterized by subsurface porosity that affects enamel’s optical properties while maintaining its surface integrity. Resin infiltration is a minimally invasive approach to managing these lesions by penetrating and sealing pores with low-viscosity resin, potentially modifying their visual appearance. In order to provide a quantifiable, objective assessment of this optical behavior, this study aimed to evaluate the modulation of color coordinates and VITA Classical shade transitions within the initial lesions immediately following infiltration with Icon Smooth Surface (DMG, Hamburg, Germany). Materials and Methods: Forty-nine extracted human premolars with naturally occurring proximal ICLs (subsurface demineralization) were selected. Lesions were assessed for fluorescence level using 655 nm laser fluorescence (DIAGNOdent pen). Reflectance spectrophotometry (SpectroShade Micro) was employed to record color coordinates in the CIELab and CIELCh spaces, alongside VITA Classical shades, before and after resin infiltration. Results: The infiltration procedure resulted in a statistically significant increase in b* (yellowness) and subsequent C* (chroma) parameters (*p* < 0.05). VITA Classical shade guide analysis showed an insignificant shift in color distribution according to value (*p* > 0.05). Conclusions: The present exploratory study provides quantitative insights into the immediate color-modifying effects of a resin infiltration procedure on natural initial proximal caries lesions, complementing the existing literature with objective CIELab/CIELCh and VITA Classical measurements. The *p*-values should be interpreted as descriptive indicators of potential trends rather than definitive statistical proof. Further research with larger cohorts and long-term follow-up is needed to validate the durability and clinical relevance of these esthetic outcomes.

## 1. Introduction

Initial caries lesions (ICLs) arise as a first dental caries manifestation in enamel, which results in an area of increased subsurface porosity. This porosity-gradient biogenic structure alters the light scattering and optical properties of the enamel while preserving its outer morphology [1].

The microscopic image under polarized light of a vertical section through enamel with such a lesion is characterized by the presence of four layers, differing in pore volume and appearance. These layers, starting from the deepest to the enamel surface, are as follows:

- Transparent layer—pore volume of 1%.
- Dark layer—pore volume of 2–4%.
- Central layer—pore volume of 5–25%. This is the largest and most porous part of the lesion.
- Superficial layer—pore volume of 1%.

For comparison, the pore volume in healthy enamel is approximately 0.1% [2,3].

Resin infiltration is a conservative micro-invasive intervention for early smooth surface caries in both primary and permanent dentitions. This approach is primarily recommended when demineralization extends through the enamel into the outer third of the dentin [4].

The microporosities within early carious lesions function as pathways for acid diffusion, bacteria and dissolved mineral. Capillary action drives monomer infiltration into these spaces, where the depth and completeness of penetration depend predominantly by total pore volume and capillary radius. However, naturally occurring lesions feature a pseudointact surface layer characterized by high mineral density and low pore volume, which restricts resin uptake. Effective infiltration requires the disruption or removal of this hypermineralized zone to expose the underlying, highly porous lesion body. Pre-treatment surface conditioning—commonly accomplished by acid etching—erodes or perforates this outer layer, thereby facilitating monomer penetration [5,6].

Dental resin used for the infiltration procedure is formulated with a low-viscosity methacrylate-based resin matrix, primarily composed of triethylene glycol dimethacrylate (TEGDMA). This low viscosity (approximately 18–20 mPa·s) ensures effective infiltration into microporosities and subsurface enamel lesions, which are typically less accessible to conventional restorative materials. Studies have demonstrated that this property facilitates lesion depth penetration up to 200–300 μm in enamel lesions [7].

A critical parameter indicating resin’s ability to infiltrate lesions is penetration coefficient (PC). With a high PC value, the resin effectively permeates capillary-like spaces within enamel and dentin. The penetration coefficient is attributed to the combination of low viscosity and hydrophilic characteristics, aided by the presence of ethanol as a solvent. This property is particularly beneficial for achieving uniform penetration into white spot lesions and early carious defects [7].

The color effect of the material is based on the refractive index (RI). For healthy enamel, the RI is 1.62. The micropores in demineralized enamel are filled with water (RI = 1.33) or, when dried, with air (RI = 1.0). Differences in this index at phase transitions cause light scattering, making initial carious lesions appear whitish to the human eye, often distinguishing them more or less clearly from surrounding healthy enamel. After infiltration, the micropores are permanently filled with resin, which has an RI of 1.46. This reduction in the difference allows light rays passing through these two phases to bend less sharply, causing the lesion to blend in color with the surrounding healthy enamel [8,9].

Tooth color is influenced by a combination of its optical properties. When light interacts with a tooth, four main phenomena occur: (1) specular transmission of light through the tooth, (2) specular reflection at the surface, (3) diffuse reflection at the surface, and (4) absorption and scattering of light within the dental tissues. Tooth color is primarily determined by the scattering of light within the tooth, where light follows irregular paths before emerging at the surface and reaching the observer’s eye. Non-white colors mainly result from absorption along these paths, which depends on the absorption characteristics of the tooth tissues. Vaarkamp et al. studied light propagation through 0.85 mm thick bars of human enamel and dentine. They found that hydroxyapatite crystals in enamel significantly contribute to light scattering, while in dentine, optical anisotropy suggested that the tubules are the primary cause of scattering. The optical scattering power of enamel was shown to increase by about three times as its mineral content decreased due to demineralization [10,11].

The majority of existing laboratory studies evaluate colorimetric parameters after infiltration using artificially induced, chemically accelerated demineralization models; the data on naturally created lesions is limited, creating a literature gap [12].

The aim of the work was to experimentally determine selective optical color properties of initial caries lesions after resin infiltration. The null hypothesis tested was that resin infiltration would induce no changes or modifications in any of the evaluated color space coordinates L*, a*, b*, C*, h*, ΔE or VITA Classical shade distribution in ordinal scale by value of the infiltrated natural enamel lesions.

## 2. Materials and Methods

**Figure 1:**
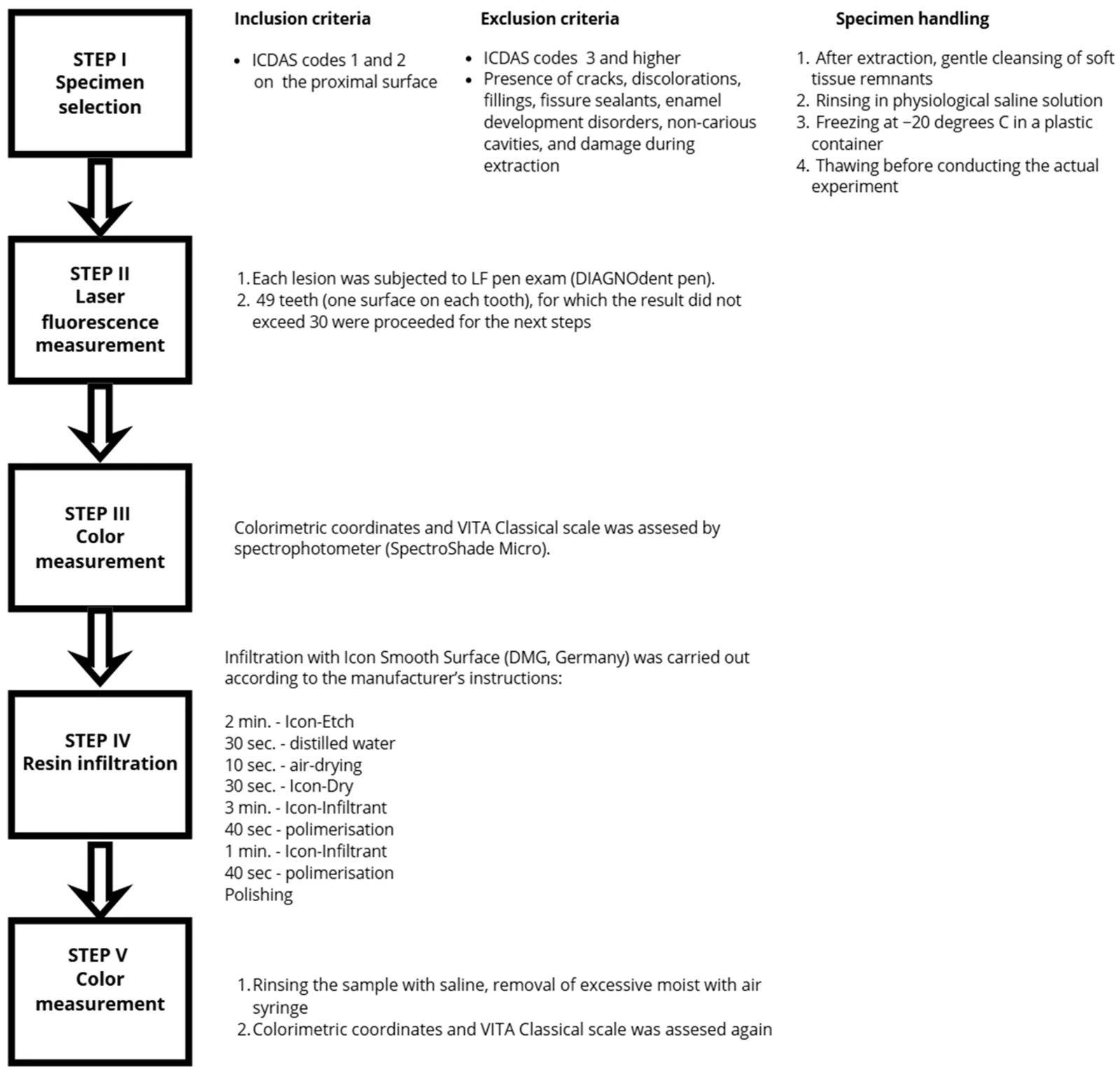
Methodology flowchart.

To evaluate the effect of the resin infiltration procedure on the optical characteristics of initial caries lesions, extracted human maxillary and mandibular permanent premolars, removed for orthodontic indications, were utilized as study samples. This selection allowed for the analysis of initial caries lesions that developed under natural oral conditions. The study received ethical approval from the Bioethics Committee at the Medical University of Lublin (No. KE-0254/54). A methodology flowchart is shown in Figure 1.

### 2.1. Experiment Procedure

The teeth examination was conducted in a dental office under dental unit lighting. Specimen selection was based on the following criteria:

#### 2.1.1. Step I—Material Selection

1. Inclusion criteria:
  - Presence of an initial carious lesion (white spot lesion with a matte surface) on wet or dried interproximal enamel, with no macroscopically detectable structural breakdown (ICDAS codes 1 and 2).
2. Exclusion criteria:
  - Macroscopic enamel breakdown or cavitation (ICDAS codes 3 to 6).
  - Presence of enamel cracks, intrinsic or extrinsic discolorations, existing restorations or fissure sealants, developmental enamel defects, non-carious lesions, or damage during extraction.

Immediately following extraction, selected teeth were cleaned of soft tissue debris, rinsed with saline, and stored in plastic containers at −20 °C until further use. Before starting the next experimental phase, specimens were thawed at room temperature for 14 h. High humidity (100%) was maintained by placing a moist cotton roll at the bottom of each container ensuring no direct contact with the teeth. Once thawed, the teeth were thoroughly cleaned using a prophylactic brush attached to a slow-speed handpiece with water, followed by a final rinse with saline. Only one surface per tooth was subjected to further parts of the study. According to previous studies, this procedure does not significantly affect red fluorescence, even over extended observation periods [13].

Prior to the main study, intra-examiner and inter-examiner reliability were assessed through the re-evaluation of 20% of the material. Intraclass Correlation Coefficients (ICC > 0.90) for continuous optical parameters and Cohen’s kappa coefficient (k = 0.88) for categorical VITA Classical shades demonstrated excellent intra- and inter-examiner reproducibility.

#### 2.1.2. Step II—Laser Fluorescence Measurement

To evaluate the lesion progression, each lesion on the smooth interproximal surfaces was scanned with DIAGNOdent pen diagnostic laser (KaVo Dental, Biberach, Germany) following the manufacturer’s protocol. The device was calibrated using a ceramic standard included in the kit. After reading each lesion twice, the maximum peak fluorescence level was recorded.

The DIAGNOdent pen is a laser fluorescence device designed for the detection of dental caries. It operates by emitting a diode laser at a wavelength of 655 nm onto the tooth surface. This laser light is absorbed by bacterial metabolites within the tooth structure, leading to the emission of red fluorescence. The device captures this fluorescence and provides a numerical reading between 0 and 99, corresponding to the severity of caries present. Higher values indicate a greater extent of decay. Subsequent testing was carried out on 49 teeth displaying DIAGNOdent readings below 30—the threshold at which operative intervention is recommended by Lussi et al. [14].

The selected and prepared teeth were then subjected to spectrophotometric examination to assess the color of the carious lesion.

#### 2.1.3. Step III—Color Assessment

Spectrophotometric measurement

The color of the carious lesions was assessed before and after treatment using the SpectroShade Micro spectrophotometer (MHT Optic Research AG, Niederhasli, Switzerland). Spectrophotometers are among the most precise instruments for color evaluation, operating by analyzing the amount of light absorbed or reflected by an object across the visible spectrum through built-in microsensors. These devices feature an independent light source with parameters closely resembling natural light [15,16].

The obtained data were converted into L*a*b* and L*C*h* color space values, as defined by the Commission Internationale d’Eclairage (CIE), and expressed as a specific shade, typically based on the VITA Classical shade guide. Before each measurement, the SpectroShade device was calibrated. The samples were tested against a black, matte background using specialized attachments provided by the manufacturer to eliminate external light interference. The tooth was fixated and the measurement was obtained by approximating the device parallelly to the tested interproximal surface to obtain the image of the lesion. After that, the lesion area was selected on the device’s screen and the measurements were read.

For the quantitative parameters in the CIELab and CIELCh color spaces, each specimen was measured twice consecutively both before and after the resin infiltration procedure. The arithmetic mean of these two readings was calculated and recorded as the final coordinate value for statistical analysis. Conversely, for the categorical VITA Classical shade determination—where numerical averaging is not possible—a majority-consensus protocol was applied. Each sample was evaluated twice; if an identical shade was obtained, it was recorded as the final value. In the event of a discrepancy between the initial two readings, a third independent measurement was performed, and the shade that appeared twice out of the three total assessments was definitively registered.

The L*a*b* color system records colorimetric parameters three-dimensionally: lightness (L*; 0 [black] to +100 [white]), green–red chromacity (a*; −[greener] to + [redder]), and blue–yellow chromacity (b*; −[bluer] to + [yellower]). The L*C*h* system is a cylindrical representation derived directly from the rectangular CIELab coordinates. The L* remains identical, the C* axis represents differences in chroma (−[duller] to + [brighter]) and h* represents differences in hue with angular values from 0 to 360 degrees. The value 0 is +a* (red), 90 is +b* (yellow), 180 is −a* (green), and 270 is −b* (blue) [15,16]. Chroma (C*) and hue (h*) are not separate spectrophotometric measurements, but they are calculated as follows:

$$C*=\sqrt{{\left(a*\right)}^{2}+{\left(b*\right)}^{2}}$$

$$h*=arctan\frac{b*}{a*}$$

Color difference (ΔE) was measured as the difference between the examined area and the closest color in the VITA Classical shade guide.

In VITA Classical scale, colors are placed in 4 groups, where the shade is defined by a letter (A, B, C, D). Within each group, there is also a number—as it increases, the color saturation increases and its brightness decreases.

#### 2.1.4. Step IV—Infiltration

Each lesion was subsequently treated with a dedicated smooth-surface resin infiltration kit (Icon Smooth Surface, DMG, Hamburg, Germany). The system includes three syringe-dispensed components and specialized applicators. According to the manufacturer’s specification, Icon-Etch (15% hydrochloric acid) removes approximately 40–50 µm of the superficial enamel layer, while Icon-Dry (99% ethanol) desiccates the surface prior to infiltration. The sealant resin (Icon-Infiltrant) is a light-cured monomer based on triethylene glycol dimethacrylate (TEGDMA) and camphorquinone as a photoinitiator. Its low viscosity, low contact angle, and high surface tension promote efficient capillary action into the lesion body [5,6,7,17]. The photoinitiator enables polymerization upon exposure to blue light (450–490 nm). The polymerization shrinkage of Icon resin is moderate, ensuring tight adaptation to the lesion walls while minimizing internal stresses that might compromise sealing. The degree of conversion (DC) has been measured at approximately 56.26 ± 10.79% under optimal curing conditions, which ensures sufficient hardness and long-term stability [18].

Resin infiltration procedure strictly adhered to the manufacturer’s guidelines. Icon-Etch was applied over the demineralized zone—extending roughly 2 mm beyond the lesion margins—and gently agitated for 2 min. The gel was then thoroughly rinsed with distilled water for 30 s and enamel surface air-dried for 10 s. Next, Icon-Dry was applied for 30 s, followed by another 10 s of air drying. To ensure sample standardization, each lesion was etched only once.

Following desiccation, the light-cured resin (Icon-Infiltrant) was applied to the lesion surface and left for 3 min. Excess material was carefully removed before polymerization for 40 s using a polymerization lamp (wavelength: 430–480 nm, light intensity: 1470 mW/cm^2^ ± 20%, Elipar DeepCure L, 3M, North Ryde, Australia). A second layer of infiltrant was then applied for 1 min; the excess was removed, and it was light-cured using the same parameters.

Final polishing was completed using a rubber cup (KENDA 0384—Ultra-fine; KENDA, Vaduz, Liechtenstein) mounted on a slow-speed handpiece. Specimens were then rinsed with a water–air spray and dried with an air syringe, in accordance with the manufacturer’s recommendations.

#### 2.1.5. Step V—Color Measurement

Right after the procedure, the sample was rinsed with saline, excessive moisture was removed with an air syringe, and color was reassessed with the spectrophotometer in the same manner as before infiltration.

### 2.2. Statistical Analysis

Prior to statistical analysis, data normality was evaluated using the Kolmogorov–Smirnov, Lilliefors, and Shapiro–Wilk tests. Because most variables deviated from a normal distribution, non-parametric methods were applied. Quantitative data were summarized using the arithmetic mean, confidence intervals, median, range (minimum and maximum), lower and upper quartile, and standard deviation. Categorical variables were presented as absolute counts and percentages. Pairwise comparisons between dependent measurements were performed using the Wilcoxon signed-rank test. The analysis included a division based on the VITA Classical scale and color components according to the CIELab and CIELCh systems. Adjustments for multiple comparisons were not applied, as each CIELab/CIELCh parameter represents a distinct, pre-planned optical dimension tested a priori, and applying overly conservative corrections would increase the risk of Type II errors in this exploratory experimental study. Exact *p*-values and effect sizes are reported to enable independent judgment of the results. A significance level of α = 0.05 was adopted for all tests. The results were calculated using Statistica 12 PL software (StatSoft, Kraków, Poland).

## 3. Results

The color of each examined sample was analyzed and described using numerical values of the parameters L*, a*, b*, C*, h*, ΔE, both before and after infiltration. The statistical analysis of the results before and after infiltration was conducted using the Wilcoxon signed-rank test (Table 1).

Statistically significant differences were found in the b* parameter values before and after infiltration. The b* values after infiltration are statistically significantly higher (Median = 21.30) than before (Median = 16.90) (*p* < 0.05).

Statistically significant differences were also found in the C* parameter values before and after infiltration. The C* values after infiltration are statistically significantly higher (Median = 21.50) than before (Median = 17.30) (*p* < 0.05).

No statistically significant differences were found for the remaining parameters (*p* > 0.05).

**Figure 2:**
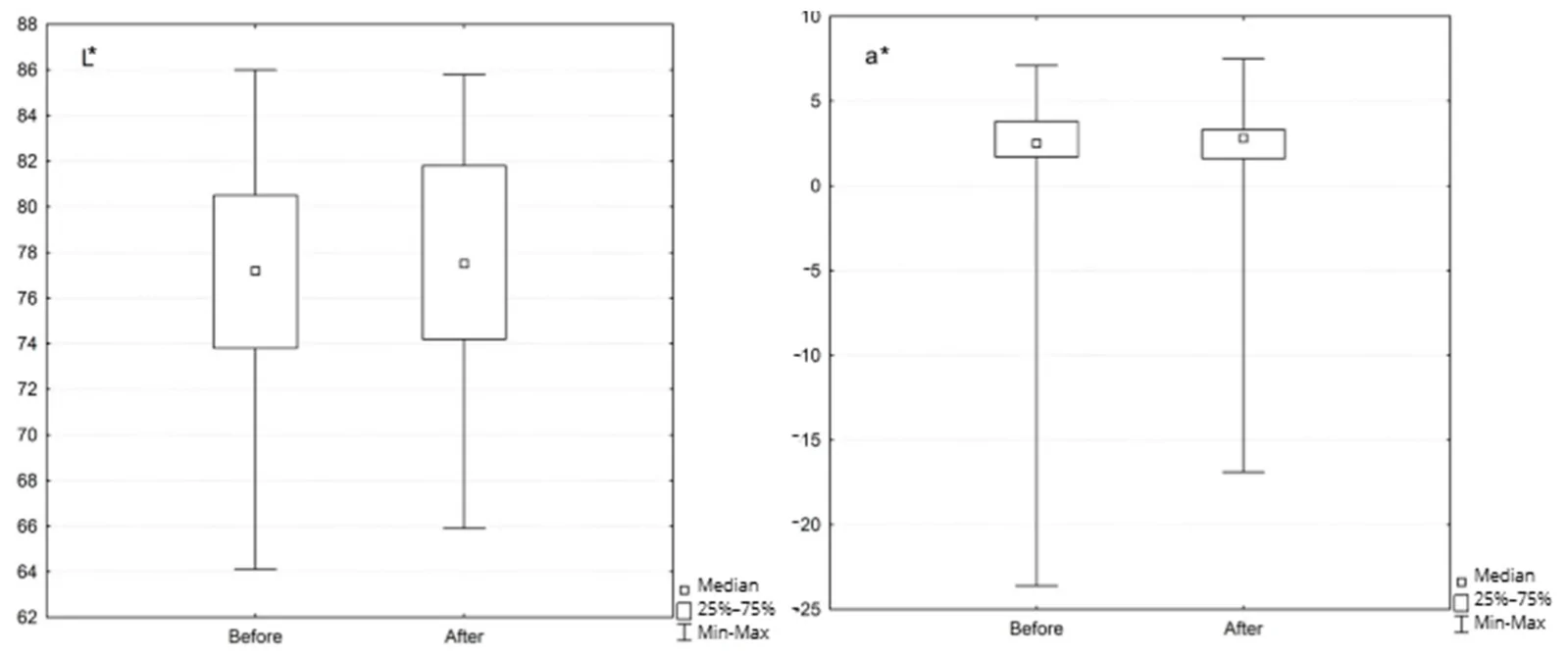

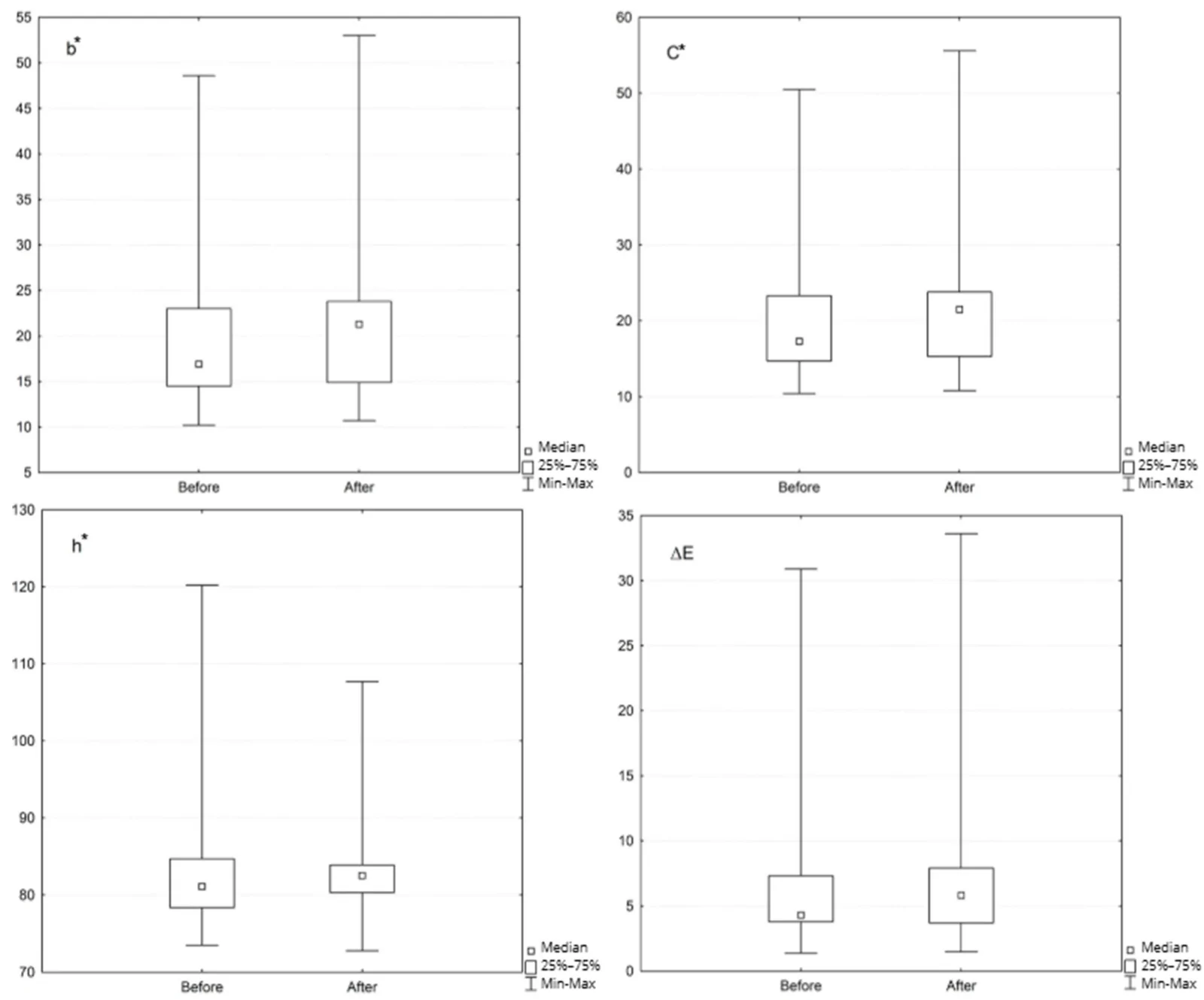
Median values of the analyzed parameters before and after the infiltration procedure.

The comparison of median values for the analyzed parameters before and after the infiltration procedure is shown in Figure 2.

**Figure 3:**
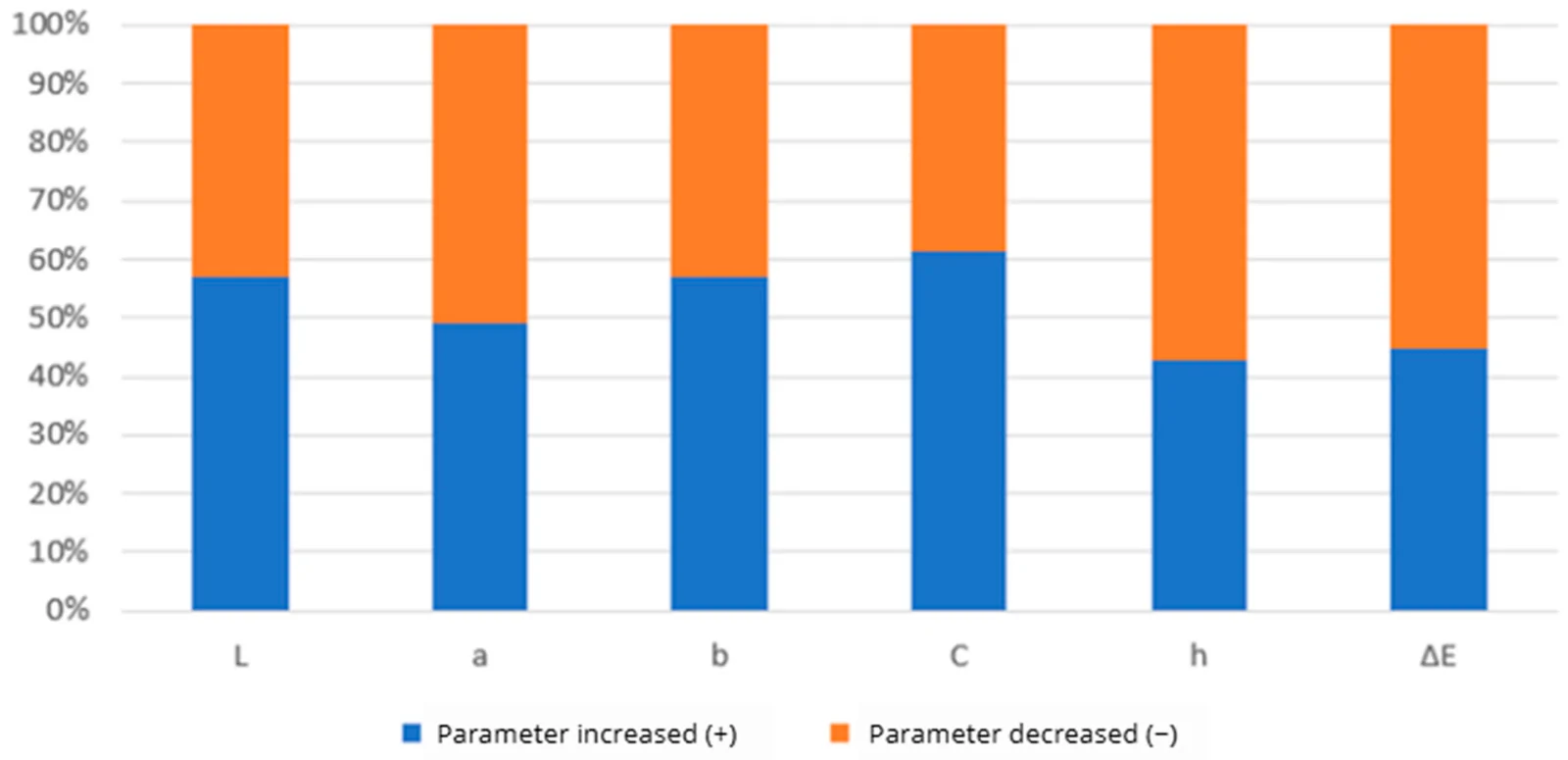
Percentage distribution of changes in the analyzed color parameters after infiltration.

Changes in the values of the analyzed parameters that occurred after the infiltration procedure were analyzed (Table 2, Figure 3). The changes were classified into two categories: increase (+) when the value of a given parameter increased, and decrease (−) when the value of the parameter decreased. For each analyzed parameter, the number of samples and their percentage distribution were calculated.

The highest discrepancy in the distribution of “increase (+)” and “decrease (−)” changes was observed for parameter C (increase or decrease in color saturation)—61.22% (*n* = 30) of “increase (+)” changes and 38.78% (*n* = 19) of “decrease (+)” changes. The lowest discrepancy occurred for parameter a (from green to red)—48.98% (*n* = 24) of “(increase)” changes and 51.02% (*n* = 25) of “decrease (−)” changes.

### VITA Classical Scale

The measurements obtained in VITA Classical scale were arranged in ordinal order by value to allow clear and consistent data presentation and statistical analysis [19,20] (Table 3).

Table 4 presents the numerical and percentage distribution of colors in the VITA Classical shade guide for the surfaces of the examined teeth, measured using the SpectroShade device before and after the infiltration treatment (Wilcoxon test, *p* > 0.05).

**Figure 4:**
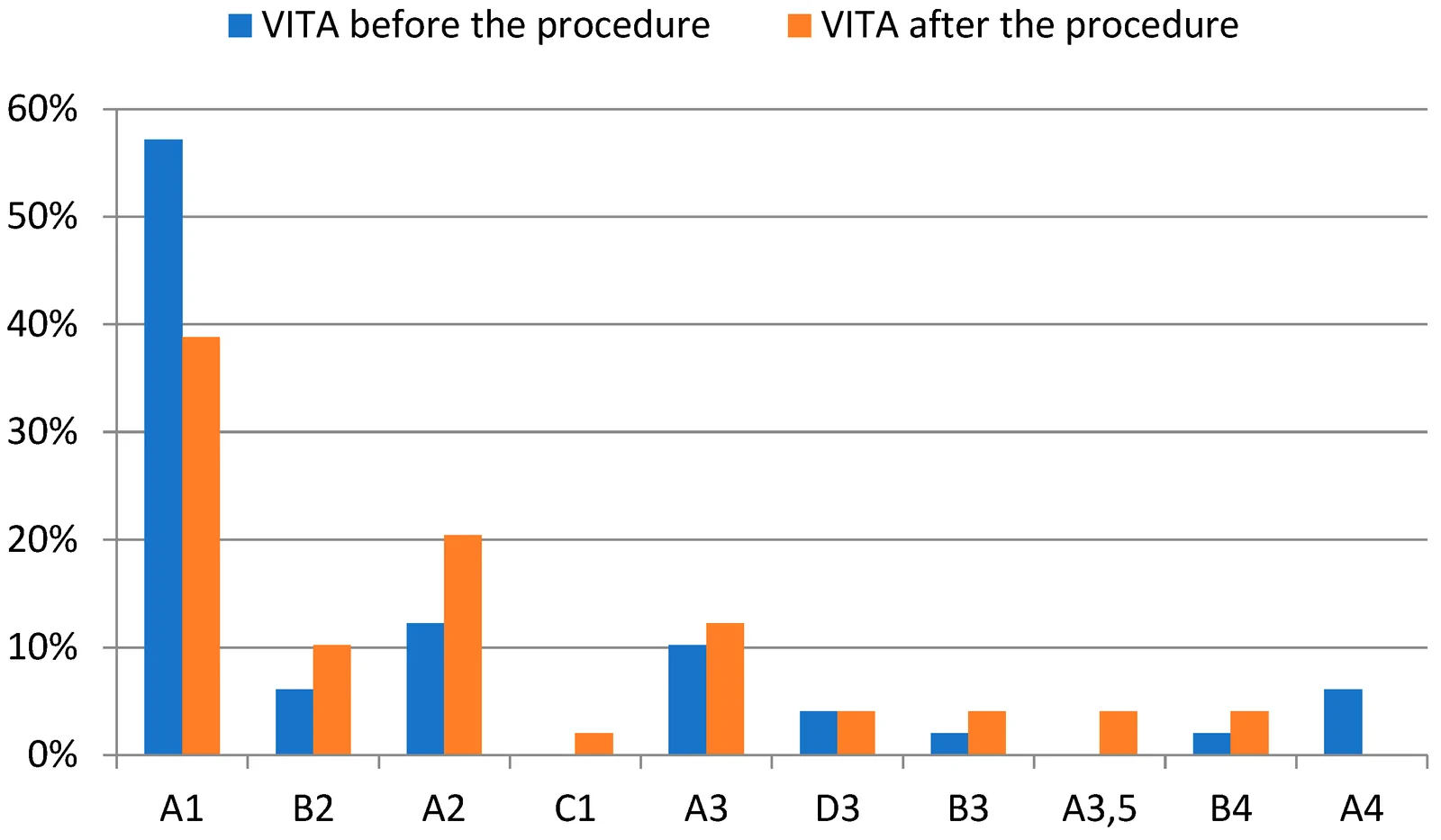
Distribution of tooth sample colors in the VITA Classical shade guide before and after infiltration treatment.

Figure 4 shows a summary of the number of samples with a given color before and after infiltration. The most samples represented the color A1, both before (*n* = 28, 57.14%) and after (*n* = 19, 38.78%) infiltration. In summary, the majority of samples were in the A group of the VITA shade guide (before: *n* = 42, 85.71%; after: *n* = 37, 75.51%), followed by the B group (before: *n* = 5, 10.20%; after: *n* = 9, 18.37%), then the D group (before: *n* = 2, 4.08%; after: *n* = 2, 4.08%), and finally the C group (before: *n* = 0; after: *n* = 1, 2.04%). This distribution indicates a shift from lighter (A1) toward slightly darker and more saturated shades after infiltration.

Table 5 summarizes changes in samples on the numerical value scale. More than half of the samples did not change color (*n* = 26, 53.06%) while 16.33% of samples (*n* = 8) shifted to colors with a smaller numerical order value and 30.61% (*n* = 15) of samples changed to colors higher on the numerical scale.

**Figure 5:**
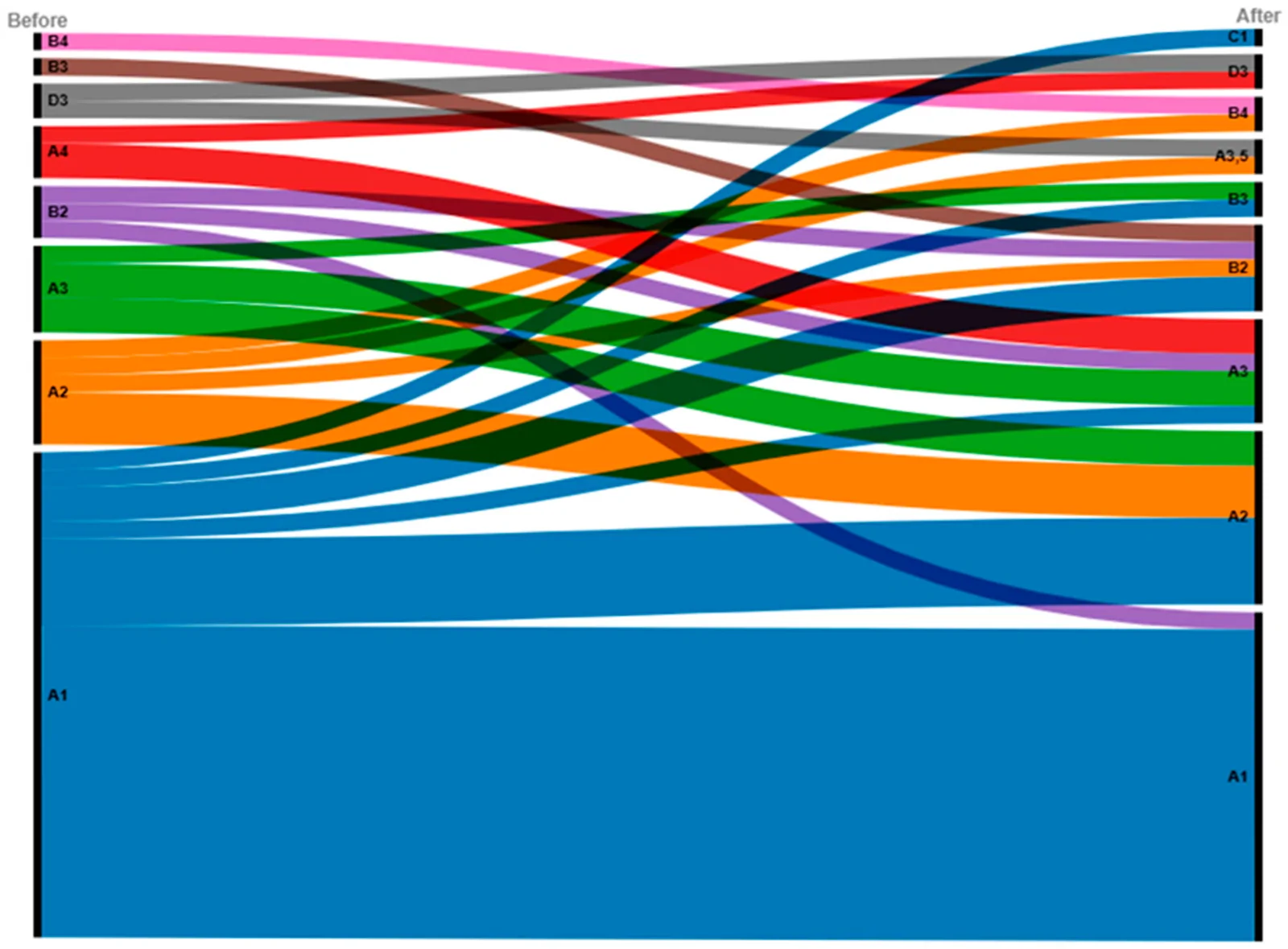
Alluvial graph of tooth sample color changes in the VITA Classical shade guide before and after resin infiltration.

The alluvial graph (Figure 5) illustrates the correspondence between pre- and post-infiltration shade categories on the VITA Classical scale. The majority of samples initially scored as A1 either remained in this category (*n* = 18) or shifted toward adjacent, slightly darker shades such as A2 (*n* = 5), A3 (*n* = 1), and B2 (*n* = 2). This trend reflects a redistribution of color values, with a reduction in the lightest shades and a movement toward hues with lower value.

## 4. Discussion

Perception of tooth color should be considered on multiple levels, including physical optics, visual physiology, visual psychology, and psychophysics. Determining tooth color is one of the more challenging aspects of restorative dentistry due to the subjective nature of visual assessment methods. Currently, several devices are available for electronically and objectively determining tooth color. These devices help eliminate subjectivity associated with color determination and increase the consistency of measurements [21,22]. The literature contains studies [23,24,25] on color changes in surfaces subjected to an infiltration procedure. In studies examining color, authors used different methodologies compared to those applied in this study, making direct comparison of results challenging. The data obtained in the analysis were converted to the L*a*b* and L*C*h* color systems as defined by the Commission Internationale d’Eclairage (CIE), using the CIELab and CIELCh systems, and also presented as specific color codes (VITA Classical). In the presented study, a statistically significant increase in the parameters b* and subsequent increase in C* (*p* < 0.05) was observed. An increase in the b* parameter indicates a greater contribution of the color yellow in the analyzed sample. A change in the C* parameter (increase in value) indicates an increase in color saturation, moving away from the point of absolute whiteness. Given the exploratory nature of this study, no formal mathematical adjustment for multiple comparisons (e.g., Bonferroni correction) was applied. Therefore, *p*-values should be interpreted as descriptive indicators of potential trends rather than definitive statistical proof.

In 2019, Kannan et al. [23] conducted an in vivo study on 240 initial carious lesions present on the surfaces of 193 teeth from 12 patients (aged 12–30 years) who had undergone orthodontic treatment with fixed braces. The study included teeth with relatively low fluorescence values (ranging from 2 to 9) measured using DIAGNOdent. The aim of the study was to examine and compare changes that would occur on the tested surfaces after the application of Icon infiltrant and a glass ionomer-based resin-modified cement Clinpro XT Varnish (3M ESPE, Pymble, Australia), which contains fluoride, calcium, and phosphorus. The patients were divided into two groups: Group 1—Icon infiltration (six patients, 102 teeth, 124 white spots) and Group 2—Clinpro XT Varnish application (six patients, 91 teeth, 116 white spots). The surfaces were assessed using the VITA Easyshade device (VITA Zahnfabrik, Bad Sackingen, Germany) at the following time points: T0—immediately before the procedure, T1—immediately after the procedure, T2—after 3 months, T3—after 6 months. In our experiment we utilized immediate-only design, so our findings can be compared to the T0 and T1 measurements. The parameters evaluated included individual color components in the CIELab scale and the ΔE parameter, defined as the color difference between the demineralized area and the surrounding healthy enamel. In the results of the study, the average L* value of the surfaces before infiltration was 73.60  ± 7.71, while after infiltration it was 78.37 ± 5.94, with the difference between the groups being statistically significant (*p* < 0.01). In the present study, the analogous value before infiltration was 77.38  ± 5.28 (Me = 77.20), while after infiltration it was 77.64  ± 4.95 (Me = 77.50), and the difference was not statistically significant (*p* > 0.05). For 28 samples (57.14%), the parameter value increased, while for 21 samples (42.86%), it decreased. An increase in this parameter indicates higher brightness of the analyzed sample, while a decrease indicates lower brightness. No statistical significance was calculated for the differences in the a* and b* parameters at various time points in the cited publication. For the a* parameter, its average value in the analyzed experiment was 0.026 ± 1.76 before infiltration and −0.29 ± 1.38 after infiltration. In the present study, these values were 1.62 ± 5.60 (Me = 2.50) before and 2.14 ± 3.71 (Me = 2.80) after infiltration, and the difference was not statistically significant (*p* > 0.05). For 24 samples (48.98%), the parameter value increased, while for 25 samples (51.02%) it decreased. An increase in this parameter indicates an increased contribution of red color in the analyzed sample, while a decrease indicates a reduced contribution and a shift toward green color. In the cited publication, the average b* parameter values were 18.39 ± 5.31 before infiltration and 21.47 ± 7.32 after the procedure, indicating an increase. In the present study, these values after infiltration (M = 20.84 ± 7.70, Me = 21.30) were statistically significantly (*p* < 0.05) higher than those before the procedure (M = 20.07 ± 8.18, Me = 16.90). For 28 samples (57.14%), the parameter value increased, while for 21 samples (42.86%) it decreased. An increase in this parameter indicates a higher contribution of the color yellow in the analyzed sample, while a decrease indicates a reduced contribution and a shift toward blue. Considering that demineralized areas are usually perceived as white compared to the surrounding healthy area, an increase in the yellow color component may likely have a beneficial effect on masking the color of the white carious spot.

In the analyzed study, the average value of the ΔE parameter, defined as the difference in color between the demineralized area before infiltration and after the procedure, was 5.68  ± 1.24, which was clinically noticeable. However, it is unclear whether this change led to the masking of the demineralized area. In the present study, the ΔE parameter was defined differently, so it cannot be directly compared to the above results.

In the methodology used, ΔE was defined as the difference between the examined area and the closest color in the VITA Classical shade guide. It is important to note that the use of a color guide in visual methods is highly subjective, which is its main disadvantage [26,27,28]. In the present study, the VITA Classical shade guide was chosen due to its widespread use and the desire to relate research results to this practical tool, which is currently one of the most commonly used for tooth color assessment [22,27]. The software of spectrophotometers, including the SpectroShade Micro device, contains CIELab coordinates for colors in the VITA Classical scale. During analysis of the area of interest, the device shows its parameters divided into L*, a*, b* components and selects the most matching shade in the VITA scale—in terms of the lowest ΔE value between the examined area and the color shade. In the study material, the mean ΔE values before infiltration were 6.71 ± 6.35 (Me = 4.30), and after infiltration 6.58 ± 5.47 (Me = 5.80). This difference was not statistically significant (*p* > 0.05). An increase in ΔE value indicates an increased color difference, while a decrease indicates a reduction. According to the adopted definition of this parameter, in this case it does not indicate how the color of the tested samples changed. However, it points to the fact that both before and after infiltration, the color of the examined areas was noticeably different from the color of the closest standard from the VITA Classical scale. It is worth noting that in the case of teeth, a clinically acceptable color match is considered to be when two areas are characterized by the parameter ΔE ≤ 3.7 [29]. Standardized laboratory conditions allow the human eye to detect a difference of ΔE = 1 [30]. In practice, this may mean that the assessment of the color of the white carious spot area and its possible change after infiltration using the VITA Classical shade guide will be unreliable due to the clinically visible discrepancy between its color and the best-matching standard. It is possible that the use of a more advanced tool, e.g., VITA 3-D Master, would allow for a more accurate shade selection to match the color from the shade guide [29].

In the analyzed material, statistically significant changes in the examined color parameters occurred in the CIELab and consequently CIELCh system. A statistically significant increase was observed for both the yellowness coordinate (b*) and chroma (C*). As described in the Materials and Methods section, C* is not an independent observation. Although these two parameters are mathematically linked, reporting both allows for a comprehensive description: the increase in b* identifies the specific directional shift toward yellow, which consequently led to a higher overall color saturation C* of the samples. We also note the marginal statistical significance of the results (0.038 and 0.048) and lack of multiple comparisons correction.

The majority of the demineralized areas examined, both before infiltration (57.14%) and after the procedure (38.78%), exhibited a color of A1 on the VITA Classical scale. Overall, within the A color group, 85.71% of samples were in this range before the infiltration, while after the procedure, this number decreased to 75.51%. The predominance of colors from the A range corresponds with studies by other authors; however, a different distribution of individual colors from this group was observed among healthy, natural teeth. Bayindir et al. [31] conducted a study using visual assessment and the VITA color guide at the University of Ohio on a sample of 359 incisors and canines from the maxilla of 120 patients. The results showed that approximately 44% of the teeth were in the A color group, with the highest proportion being color A2 (about 11%). In 2012, Indian researchers examined the maxillary incisors of 400 patients using the VITA Easyshade spectrophotometer. The patients were grouped by gender and age. In the 15–25 age group, the most commonly observed color was A1 (42% of women and 56% of men), while in older age groups, A3 predominated (ranging from 32% to 38%) [32]. A 2015 study at a university in Sudan showed that most (78.50%) of the examined maxillary central incisors, assessed using the VITA Easyshade spectrophotometer from 226 patients, were in the A color range. Among them, the most frequent color was A3 (36.10%), followed by A2 (27.30%) and A1 (11.50%) [33]. This demonstrates that the majority of the demineralized changes in the present study, both before and after infiltration, exhibited a color with lower saturation and higher brightness compared to the average in the general population.

The present study provides quantitative insights into the immediate color-modifying effects of resin infiltration on natural initial proximal caries lesions, complementing existing literature studies with objective CIELab/CIELCh and VITA Classical measurements. Several limitations must be acknowledged. The treatment was evaluated as a complete clinical procedure; the observed optical modifications represent the net effect of the combined protocol—including surface conditioning (acid etching and ethanol dehydration)—rather than the isolated physical impact of the resin alone. There was no tracking of donors’ age, sex and tooth position (maxillary, mandibular). The experimental design did not fully replicate the oral environment; only immediate post-infiltration outcomes were assessed. This immediate-only protocol was adopted to ensure standardization of the laboratory conditions. Moreover, no separate control group of untreated or alternatively treated lesions was included; instead, a within-subject design was applied, which enabled precise evaluation of changes but restricted comparison with other treatment modalities or natural progression. Also, the adjacent healthy enamel was not examined. The study did not adopt different caries diagnostic methods (i.e., histological validation, micro-CT etc.). Colorimetric analysis could be influenced by black background and sample polishing. In our study, sample size was not calculated. The number of samples used was determined by specimen availability meeting strict inclusion criteria; future studies should employ prospective power calculations.

Future research could focus on prospective in vivo studies with larger, more heterogeneous patient cohorts, extended follow-up, and incorporation of patient-reported outcome measures to verify the durability and clinical relevance of esthetic effects. Innovative directions may include the development of bioactive or nanofilled resin infiltrants (e.g., incorporating bioactive glass or nanohydroxyapatite) that combine esthetic masking with remineralizing potential [34,35], as well as the use of advanced imaging modalities such as hyperspectral reflectance or AI-assisted analysis for sensitive long-term monitoring and calculating differences using more specific tools to assess differences (CIEDE2000 (Δ*E*_00_)) [36].

## 5. Conclusions

Based on this exploratory in vitro model study, the following conclusions can be drawn:

1. Resin infiltration procedure of natural initial carious lesions on interproximal tooth surfaces resulted in non-significant change in color distribution on the VITA Classical scale (*p* > 0.05).
2. In the studied sample, the infiltration procedure led to an increase in the yellowness coordinate (*b) and subsequent color saturation parameter (*C) on the CIELab and CIELCh scales (*p* < 0.05).

## Figures and Tables

**Table 1:** Characteristics of color parameters of the examined surfaces before and after the infiltration procedure using the SpectroShade device, along with statistical analysis of the results.

Parameter	Examination	M	−95% CI	+95% CI	SD	M_e_	Min.	Max.	Q_1_	Q_3_	
L	before	77.38	75.51	79.26	5.28	77.2	64.1	86.0	73.8	80.5	Z = 0.063 *p* = 0.950 r = 0.009 d = 0.018
	after	77.64	75.88	79.40	4.95	77.5	65.9	85.8	74.2	81.8	
	difference	0.25	−0.78	1.29	2.92	−0.4	−4.0	8.4	−2.0	2.2	
a	before	1.62	−0.37	3.61	5.60	2.5	−23.6	7.1	1.7	3.8	Z = 0.439 *p* = 0.660 r = 0.063 d = 0.126
	after	2.14	0.83	3.46	3.71	2.8	−16.9	7.5	1.6	3.3	
	difference	0.52	−1.05	2.09	4.42	0.0	−2.9	24.2	−0.9	0.5	
b	before	20.07	17.16	22.97	8.18	16.9	10.2	48.6	14.5	23.0	**Z = 2.077** ***p* = 0.038** r = 0.297 d = 0.622
	after	20.84	18.11	23.57	7.70	21.3	10.7	53.0	14.9	23.8	
	difference	0.77	−1.53	3.07	6.49	2.0	−29.5	8.8	−0.5	3.9	
C	before	20.60	17.44	23.77	8.93	17.3	10.4	50.5	14.7	23.3	**Z = 1.996** ***p* = 0.048** r = 0.285 d = 0.595
	after	21.11	18.26	23.96	8.04	21.5	10.8	55.6	15.3	23.8	
	difference	0.51	−2.14	3.16	7.47	1.2	−35.9	8.7	−0.5	3.7	
h	before	83.00	79.88	86.13	8.81	81.1	73.5	120.2	78.4	84.7	Z = 1.063 *p* = 0.288 r = 0.152 d = 0.308
	after	82.77	80.75	84.78	5.69	82.5	72.8	107.7	80.3	83.9	
	difference	−0.24	−2.58	2.10	6.60	1.1	−33.4	6.9	−1.4	2.5	
ΔE	before	6.71	4.46	8.97	6.35	4.3	1.4	30.9	3.8	7.3	Z = 1.328 *p* = 0.184 r = 0.190 d = 0.387
	after	6.58	4.64	8.52	5.47	5.8	1.5	33.6	3.7	7.9	
	difference	−0.13	−1.82	1.56	4.76	0.4	−24.7	5.4	−0.8	1.6	

M = mean, CI = confidence interval, SD = standard deviation, M_e_ = median, Min. = minimum, Max. = maximum, Q_1_ = first quartile, Q_3_ = third quartile, r = Wilcoxon signed-rank test effect size, d = Cohen’s effect size calculated based on the r value. Statistically significant results are shown in bold.

**Table 2:** Numerical and percentage distribution of changes in the values of analyzed color parameters after infiltration.

Color Parameters	Parameter Increased (+)		Parameter Decreased (−)	
	*n*	%	*n*	%
L	28	57.14%	21	42.86%
a	24	48.98%	25	51.02%
b	28	57.14%	21	42.86%
C	30	61.22%	19	38.78%
h	21	42.86%	28	57.14%
ΔE	22	44.90%	27	55.10%

**Table 3:** VITA Classical scale arranged in ordinal order by decreasing value.

VITA Shade	B1	A1	B2	D2	A2	C1	C2	D4	A3	D3	B3	A3,5	B4	C3	A4	C4
Numerical order	1	2	3	4	5	6	7	8	9	10	11	12	13	14	15	16

**Table 4:** Colors in the VITA Classical shade guide for the examined surfaces before and after infiltration.

	VITA Before the Procedure		VITA After the Procedure	
	*n*	%	*n*	%
A1	28	57.14	19	38.78
B2	3	6.12	5	10.20
A2	6	12.24	10	20.41
C1	0	0.00	1	2.04
A3	5	10.20	6	12.24
D3	2	4.08	2	4.08
B3	1	2.04	2	4.08
A3,5	0	0.00	2	4.08
B4	1	2.04	2	4.08
A4	3	6.12	0	0.00
Z = 1.049, *p* = 0.294, r = 0.183, d = 0.372				

**Table 5:** Changes in VITA Classical colors according to value order.

VITA Classical Changes	*n*	%
Higher value	8	16.33
No change	26	53.06
Lower value	15	30.61

## Data Availability

The data presented in this study are not publicly available due to legal restrictions. Anonymized data may be made available from the corresponding author upon reasonable request and with appropriate approvals.
